# Pilot Study on QTc Interval in Dogs Treated with Domperidone

**DOI:** 10.3390/vetsci11010039

**Published:** 2024-01-18

**Authors:** Giulia Donato, Tiziana Caspanello, Massimo De Majo, Marisa Masucci, Diego Iannelli, Silvia Santoro, Alessandra Caprì, Nicola Maria Iannelli, Maria Grazia Pennisi

**Affiliations:** 1 Department of Veterinary Science, University of Messina, 98168 Messina, Italy; giudonato@unime.it (G.D.); tiziana.caspanello@studenti.unime.it (T.C.); mdemajo@unime.it (M.D.M.); nicola_iannelli@libero.it (N.M.I.); mariagrazia.pennisi@unime.it (M.G.P.); 2 Clinica Veterinaria Camagna–VetPartners, 89124 Reggio di Calabria, Italy; diegoiannelli@hotmail.com (D.I.); silviasantorovet@gmail.com (S.S.); alessandracapri@hotmail.com (A.C.)

**Keywords:** domperidone, adverse side effect, dog, ECG, QT interval, QTc interval, cardiac arrythmia, creatinine, electrolytes

## Abstract

**Simple Summary:**

In humans, a pro-arrhythmic side effect of domperidone is caused by prolonged QT intervals. We evaluated this risk in dogs by measuring the corrected QT (QTc) intervals in 17 dogs receiving a four-week treatment. Electrocardiogram and blood parameter concentrations of creatinine, urea nitrogen, sodium, potassium, and chloride were evaluated seven days before the start and on the last day of therapy. In two dogs, the QTc interval was measured at four different times on the first day of treatment: before, 2 h, 3 h, and 12 h after administration of the drug. The 17 dogs showed a significant increase in QTc and chloride concentrations, but QTc post-treatment slightly exceeded the upper reference limit only in one dog, and the electrolyte concentrations were always within the reference range. Conversely, creatinine concentrations decreased significantly after treatment. In the two dogs monitored during the first day of treatment, QTc measures were always within the reference range. Domperidone administration caused a slight prolongation of QTc interval, and we suggest that further studies should be made for a risk assessment of the drug in dogs with cardiac diseases, electrolytic imbalances, and those under treatment with drugs that increase QT interval or compete with domperidone metabolism.

**Abstract:**

Domperidone is used as an immunomodulatory drug for *Leishmania infantum* infection and disease in dogs. However, a pro-arrhythmic side effect, caused by prolonged QT intervals, is reported in humans. This pilot study evaluated the corrected QT (QTc) interval in dogs treated with domperidone for preventive or therapeutic management of leishmaniosis. The electrocardiogram and blood concentration of creatinine, urea nitrogen, sodium, potassium, and chloride were evaluated seven days before the start and on the last day of therapy in 17 dogs receiving domperidone for four weeks. In two dogs, the QTc interval was measured before and 2 h, 3 h, and 12 h after administration of the drug on the first day of treatment. After treatment, QTc measures and chloride concentrations increased significantly, although the QTc value slightly exceeded the upper reference limit only in one dog, and chloride concentrations were always normal. Creatinine concentrations significantly decreased after therapy. In the two dogs monitored at different times on the first day of treatment, QTc values were always normal. Domperidone caused a slight prolongation of QTc interval, and further studies should be made for a risk assessment in dogs with cardiac diseases, electrolytic imbalance, and in those receiving drugs increasing QT interval or competing with domperidone metabolism.

## 1. Introduction

Domperidone is a peripheral dopamine 2-receptor (DA-2) antagonist with different pharmacologic properties, and it is largely used in both human and veterinary medicine. In dogs, after oral administration, domperidone reaches peak plasma levels approximately two hours after administration and has a mean elimination half-life of 3.3 ± 0.4 h [1]. In humans, the prokinetic and antiemetic properties of domperidone are exploited in gastroenteric disorders, while the stimulation of prolactin secretion allows for the use of this drug for increasing breast milk production [2,3,4]. Furthermore, the rise of serum prolactin is responsible for the enhancement of the non-adaptive immune response and possibly contributes to adaptive cell-mediated immunity. These immunomodulating effects of domperidone are used in dogs for the immunoprophylaxis of *Leishmania infantum* infection and the immunotherapy of mild forms of canine leishmaniosis (CanL) [5,6,7,8].

However, it is reported that the drug can prolong the QT interval in the electrocardiographic (ECG) trace by blocking the rapid component of the delayed rectifier potassium current (IKr) through the human ether-a-go-related gene (hERG) ion channels, which are responsible for restoring membrane potential after each cardiac action potential [9,10,11,12,13,14].

The possible cardiac adverse effects of domperidone have also been investigated in an in vitro study using a neonatal rat ventricular cardiomyocyte model. This experimental model demonstrates that domperidone can also prolong the QT interval by inhibiting cardiac Na^+^ channels [15]. Potential cardiac adverse events due to the use of domperidone, with a delay in cardiac repolarization, have been reported in human medicine [9,10,11,12,13,14]. The risks of severe and potentially fatal cardiac arrythmias in association with domperidone are known in humans [16], particularly when administered at doses >30 mg/day [2]. Moreover, prolonged QT intervals have been described when domperidone was administered in patients with concurrent electrolytic disorders (e.g., hypokalemia, hypomagnesemia, hypocalcemia) and/or some risk factors (e.g., older age, female sex, sleep apnea, complete atrioventricular block, dilated cardiomyopathy, and bradycardia) [11], or in association with other drugs that directly increase the QT interval or, indirectly, compete with domperidone metabolism and elimination (cytochrome P450-3A4 inhibitors) [12,17,18,19].

To date, the effect on the QT interval and the proarrhythmic potential of domperidone have not been investigated in veterinary medicine, and the risks of administering therapeutic doses of domperidone in association with other QT-prolonging drugs have been pointed out [20].

In ECG traces, the QT interval is measured from the beginning of the QRS complex to the end of the T-wave. Since cardiac repolarization adapts to heart rate and heart rate and QT interval have an inverse relationship, a better way to calculate the QT interval is to correct the value measured based on heart rate (QTc). This correction enables comparison between measures associated with different heart rates and reference values, so it is preferable to use the QTc value rather than the simple QT measure [21].

The effect of domperidone on slowing the progression of chronic kidney disease in dogs affected by the clinical disease caused by *L. infantum* has been previously investigated [7,22]. A significant decrease in serum creatinine [7] and symmetric dimethylarginine (SDMA) [22] concentrations was reported. Both of these studies followed up on treated dogs for several months, although they were limited by the small numbers of dogs evaluated [7,22].

The aim of this pilot study was to make a small-scale evaluation of QTc intervals from ECG tracing of dogs treated with a domperidone oral formulation according to the dosage indicated by the drug manufacturer. Selected blood parameters were also studied, with the additional aim of investigating the relationships between electrolyte concentrations and QTc measures and studying the influence of domperidone on serum creatinine values.

## 2. Materials and Methods

### 2.1. Study Design

A prospective, observational pilot study (study 1) was performed from September to November 2021 in Calabria (Southern Italy). All treatment, housing, and care of the animals were in full accordance with the guidelines of the Declaration of Helsinki and approved by the Ethics and Welfare Committee of the Department of Veterinary Medicine, University of Messina (UniME) (protocol number: 063_2021). Apparently healthy adult dogs routinely evaluated at a veterinary practice (Centro Veterinario San Rocco, Oppido Mamertina, Reggio Calabria, Italy) and a dog shelter (Canile Comunale di Mortara, Reggio Calabria, Italy) were included in the study. The dogs were enrolled after the signature of written informed consent. Dogs were excluded from the study in cases of pregnancy, history of cardiovascular diseases diagnosed, presence of signs compatible with any cardiovascular illness, and current administration of any other drugs apart from domperidone. All enrolled dogs were tested for anti-*L. infantum* antibodies by quantitative immunofluorescence antibody test (IFAT) and received a registered domperidone formulation (Leisguard^®^, 0.5 mg/kg PO q24 h) for four weeks for preventative [23] or therapeutic management of CanL [5,8]. The dogs were clinically evaluated the week before the start and on the last day of domperidone treatment. During the clinical evaluation, signalment, clinical history, and physical examination data were registered. At the end of both clinical evaluations, a blood sample and an ECG tracing were obtained from each dog.

On the first day of treatment, the ECG traces of two dogs have been additionally monitored before the drug administration (T_0_), after 2 h (T_2_), 3 h (T_3_), and 12 h (T_12_) after the drug administration (study 2). One of the dogs in study 2 received quarterly preventive treatments with domperidone and had been previously enrolled in study 1 during a previous cycle of treatment.

### 2.2. Sampling Procedures, Clinicopathological Evaluation, and Electrocardiogram Recording

Two milliliters of blood were collected from each dog, and one milliliter was placed into a lithium heparin tube and stored at +4 °C until used for the evaluation of blood urea nitrogen (BUN), creatinine (Cr), sodium (Na), potassium (K), and chloride (Cl) (Stat Profile Prime Plus^®^ VET Analyzer, Nova Biochemical, Milan, Italy). The remaining blood was used to obtain serum after clotting in a dry tube and centrifugation to perform *L. infantum* IFAT.

A computer-based ECG three-minute recording was obtained (Easy ECG Pocket, Ates Medica Device, Verona, Italy). For the ECG trace recordings, dogs were placed in a standing position using a standard technique with surface electrodes made of alligator clips attached to the skin at the level of the olecranon apophyses and over the patellar ligaments [24]. Rubbing alcohol was used to maintain electrical contact between electrodes and the skin. Heart rate was obtained from the ECG tracing with the instantaneous frequency method, which consists of dividing the number of milliseconds in a minute (60.000) by the mean of the minimum and maximum RR intervals measured in 60 s. The heart rate range was set at 70–160 bpm for standard-sized dogs and 60–140 bpm for giant-sized dogs [25]. The QT and RR intervals were measured in lead II from tracings printed on graph paper at a speed of 50 mm/s and a gain of 10 mm/mV. The definition of the end of the T-wave has been standardized by using the tangent method. In detail, the end of the T waves was considered at the point of interception of a tangent line extrapolated in the T wave at the point of maximum descending slope to the isoelectric baseline [26]. The pre-treatment (pre-QTc) and post-treatment (post-QTc) QTc values were calculated by the logarithmic QT correction formula. The formula was: QTc = log600 × QT/log RR, with the QT interval and the previous RR interval expressed in milliseconds, and the final value was the mean of the QTc values measured in 60 s [21]. ECG examinations and QTc measurements were performed by a single operator to avoid inter-operator variability. Also, the operator blindly read twice the ECG traces and calculated the QTc measurements in order to evaluate intra-operator variability.

### 2.3. Detection of Anti-Leishmania Infantum Antibodies

Anti-*L. infantum* antibodies (IgG) were detected by IFAT using *L. infantum* (strain MHOM/IT/80/IPT1) antigen slides produced by Centro di Referenza Nazionale per le Leishmaniosi (C.Re.Na.L., Palermo, Italy) and rabbit anti-dog IgG (anti-dog IgG-FITC, Sigma Aldrich, St. Louis, MO, USA). The manufacturer’s protocol was followed, and the endpoint titer of the positive samples was determined by preparing PBS (phosphate-buffered saline) in serial two-fold dilutions of serum starting from a cut-off dilution of 1:80 [27].

### 2.4. Statistical Analysis

Statistical analysis was performed using GraphPad Prism version 7.0 for Windows (GraphPad Software, San Diego, CA, USA). The distribution of continuous variables was evaluated by the D’Agostino–Pearson omnibus normality test, and descriptive statistics were performed for all the evaluated variables.

According to data distribution, a paired *t*-test or Wilcoxon matched-paired signed rank test was used to compare the pre- and post-treatment values of QTc, BUN, Cr, Na, K, and Cl. Spearman’s Rho test was used to measure the strength of the correlation between QTc and the other variables examined. A Fisher’s exact test was performed to evaluate the association between changes in QTc, heart rate, and blood parameter values and the domperidone treatment.

A paired *t*-test and Bland-Altman method comparison were used to evaluate intra-operator variability.

The significance threshold for all tests was set at *p* < 0.05.

## 3. Results

### 3.1. Dog Population Demographic and Clinical Data

A total of 17 dogs (nine owned and eight shelter dogs) were included in study 1. Most of the subjects were crossbred (n = 10), and five different dog pure breeds were represented: Siberian husky (n = 2), Caucasian shepherd dog (n = 2), Italian pointer (n = 1), American Staffordshire terrier (n = 1), and English pointer (n = 1). Male sex (n = 13) was more prevalent than female sex (n = 4). The age of dogs ranged from 24 to 108 months, with a mean age of 56.4 months (SD = ±21.6 months).

All enrolled dogs were evaluated as clinically healthy on physical examination. Their body weight ranged between 15 and 40 Kg (mean = 23 kg, SD = ±6.8 kg). A total of 15 medium-sized and 2 giant-sized dogs were included. Six dogs tested antibody-negative for *L. infantum* and followed a prophylactic course of domperidone treatment. Eight dogs were antibody positive with low titers (titer 80, n = 2; titer 160, n = 4; titer 320, n = 2), and three dogs were high *L. infantum* positive (titer 1280, n = 1; titer 2560, n = 2). The antibody-positive dogs received domperidone as a therapeutic measure. Data about the dogs enrolled in study 1 are reported in Table 1.

Dog 1.9 was also enrolled for the study 2 (dog 2.1). The other dog included in study 2 (dog 2.2) was a cross-breed female dog, aged 13 months and weighing 18 kg. Both dogs were evaluated clinically healthy on physical examination.

### 3.2. ECG Tracing and Blood Parameter Changes after Domperidone Treatment

Regarding the intra-operator variability evaluation, there were no significant differences between the corrected QTc values obtained in the repeated measurements, both for pre-treatment and for post-treatment ones. For the pre-treatment and post-treatment values, biases of −3.122 ms and −3.273 ms, SD bias of 12.36 ms and 11.27 ms, and 95% limits of agreement from −27.34 ms to 21.10 ms and from −25.36 ms to 18.81 ms were obtained, respectively.

#### 3.2.1. Study 1

Two dogs had a sinus tachycardia (Table 1: dogs 1.7 and 1.15), one crossbreed dog had 250 bpm recorded after treatment (Table 1: dog 1.7), and one Caucasian shepherd dog (Table 1: dog 1.15) had 159 bpm before treatment; 16 dogs showed a regular sinus rhythm, and only one dog had a respiratory sinus arrhythmia (Table 1: dog 1.9) on both pre- and post-treatment evaluations. The width, height, and morphology of P, QRS, and T waves were normal in all dogs. All dogs had QTc values within the reference range before the treatment, and one dog with a borderline high value of pre-QTc (240 ms) had a QTc of 240.4 ms after domperidone therapy (Table 1: dog 1.14). Post-treatment QTc duration was shorter than pre-treatment in four dogs, and 12 dogs had significantly longer QTc duration after treatment (paired *t*-test, *p* = 0.0292). A negative correlation was found between QTc values and heart rate (Spearman’s Rho test, *p* = 0.0034; *r_s_* = −0.48888).

#### 3.2.2. Study 2

**Figure 1:**
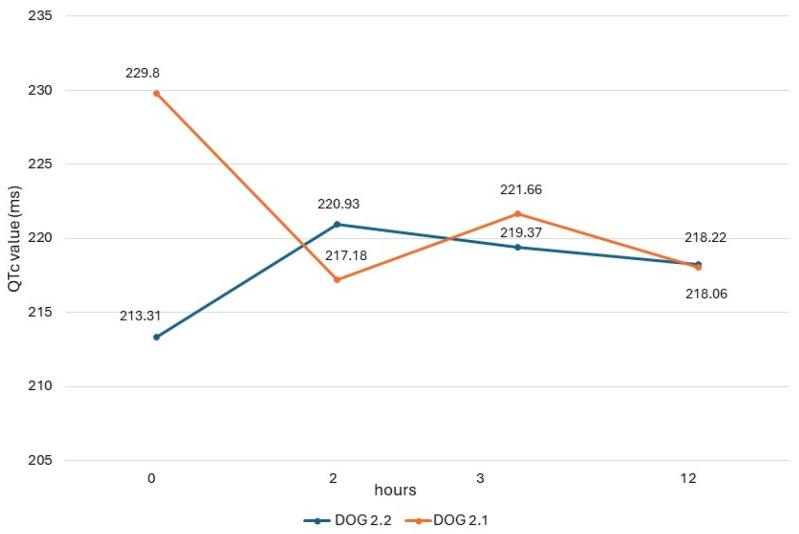
QTc values obtained in dogs of study 2 with ECG recording obtained before administration (0) and 2 h, 3 h, and 12 h after administration of the drug.

**Figure 2:**
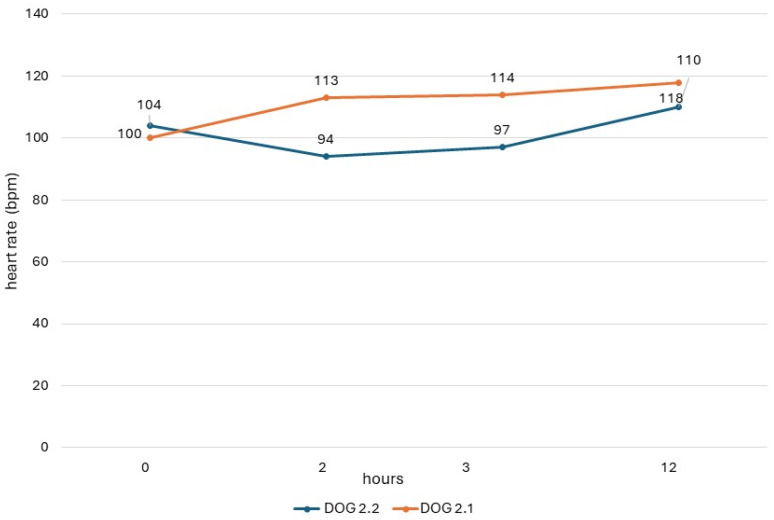
Heart rate of dogs in study 2 with ECG recording obtained before administration (0) and 2 h, 3 h, and 12 h after administration of the drug.

The QTc values obtained in dogs from study 2 (dogs 2.1 and 2.2) are shown in Figure 1, and their heart rate values are reported in Figure 2. In both dogs, there was a respiratory sinus arrhythmia present.

### 3.3. Clinicopathological Evaluation

Significantly higher Cl concentrations (paired *t*-test, *p* < 0.0001; Fisher’s exact test, *p* = 0.0445, OR = infinity, 95% CI = 1.069 to infinity) were found compared to the pre-treatment. Conversely, Cr concentration values were significantly lower (paired *t*-test, *p* = 0.0014) after treatment (Table 2).

The values of the studied blood parameters were within the reference range in all dogs both before and after treatment, except for BUN in study 1, with low outliers in five dogs before treatment and seven after treatment (Table 1).

## 4. Discussion

Domperidone oral suspension is licensed in some European countries (Spain, Portugal, Italy, Greece, and Cyprus) [28] for the prevention and treatment of CanL, and its use has been increasingly spreading in recent years [6,8,29]. Adverse effects due to domperidone administration in dogs are mild and rare [6,29]. Among them, diarrhea can be a self-limiting adverse side effect [8], while mammary gland hyperplasia with increased milk production and other disorders (e.g., lethargy, appetite loss, abdominal pain, and emesis) normally disappear after drug discontinuation [6,8]. The effect of domperidone on QT interval prolongation and possible cardiac toxicity has been extensively studied in human medicine [2,9,10,11,12,13,14,16,17,18,19,30], and the use of the drug has been restricted as a result by the European Medicines Agency in 2014 [28]. The possibility of a similar adverse effect in dogs has been previously pointed out [20], but it has not been investigated before this pilot study.

The QT interval represents the measure of time between the onset of ventricular depolarization and the completion of ventricular repolarization [31]. Therefore, a prolonged QT interval with a delay in ventricular repolarization can be responsible for the development of arrhythmias (e.g., ventricular fibrillation and torsade de pointes) [31]. Since the QT interval is influenced by changes in heart rate, some formulas have been developed to correct the length of the QT interval, defined as QTc, in relation to heart rate or to the RR interval [32]. In this study, we found significantly higher post-QTc values, but still within the physiological range. Only one dog (Table 1: dog 1.14) showed a post-QTc value just above the upper limit of the reference range and only slightly higher than pre-treatment values (0.16%). In dog 1.14, we observed a prolongation of QTc after the four-week course of treatment (240.4 vs. 240 ms). However, the post-QTc prolongation was influenced by the higher post-treatment heart rate observed (108 vs. 96 bpm). Furthermore, the post-treatment increase in QTc was observed in 76.5% of the examined animals, while the remaining 23.5% of dogs had lower post-treatment values.

ECG measurements of study 2, obtained before the drug administration (T_0_), at the maximum peak time (T_2_), and half-life time (T_3_) of an oral dose of domperidone, showed different trends of QTc values in the two dogs, similarly to results seen in the dogs of study 1. In dog 2.2, there was an increase in post-treatment values, which, however, never exceeded the reference range. On the contrary, in dog 2.1, the post-treatment values were lower than the basal value. This same dog presented higher post-treatment values in study 1, when it had been monitored during a previous cycle of preventive drug administration. These different trends could be due to different causes, including the intrinsic variability of QTc due to the many factors influencing this ECG measure [33].

All studied dogs received the recommended dose and duration of domperidone administration, and we found a prolongation of QTc in 75% of dogs receiving a standard treatment. Nevertheless, this finding had no clinical significance, as none of the dogs showed arrhythmias or other cardiac side effects, and QTc remained within the reference range in almost all dogs. On the other hand, this finding suggests that further studies should be considered to evaluate the safety of domperidone administration in patients affected by heart diseases, electrolytic disorders, or those under treatment with drugs directly responsible for a QT interval increase or that have strong cytochrome P450 3A4 (CYP3A4) inhibitory activity [12,17,18,19]. For instance, some antiarrhythmic, antihistamine, antimicrobial, psychotherapeutic, and prokinetic drugs can prolong the QT interval in dogs and cats [20,34]. CYP3A4 is responsible for domperidone hepatic metabolization, but the interaction with drugs that inhibit this enzyme, such as ketoconazole, itraconazole, erythromycin, and fluoroquinolones, may increase domperidone concentration with a higher risk for cardiotoxicity [20]. Furthermore, it is important to consider the kinetics of these drugs: if they are similar to that of domperidone, their peak would be reached simultaneously, and the risk of side effects would be greater if they were administered at the same time. On the other hand, the concurrent administration of CYP3A4 inducers may affect the pharmacokinetics of domperidone by decreasing its serum concentration and mean residence time and reducing the efficacy of the drug [35].

In addition to the above-mentioned influence of some drugs on the QT interval and heart rate, other factors can play a role in these changes, such as some electrolyte imbalances [36,37,38,39,40]. Interestingly, we found significantly higher values of Cl after domperidone treatment, although no dogs had values above the upper limit of the reference range. It is reported that conditions of hypokalemia [36], hypocalcemia [37], and hypomagnesemia [9] can determine a prolongation of the QT interval. Conversely, hypercalcemia [39] and hyperkalemia [40] can determine a shortening of the QT interval. In the present study, the values of kalemia were always within the reference range, and correlations between values of QTc and concentrations of electrolyte evaluated were not found.

We decided to record ECGs with dogs in a standing position instead of standard right lateral recumbency, with the aim of avoiding forcing the animals into this latter body position and obtaining their best adherence to the manipulations needed. However, Hertzer et al. (2022) [41] recently evaluated the effects of some variables on ECG intervals in 60 healthy dogs, with the aim of comparing real-life situations to standard recommendations. ECG interval measures of dogs, including the QT interval, did not differ in standing position compared to the standard right lateral recumbency using a six-lead ECG [40]. Therefore, the ability to measure QT intervals with the method used in the present study is validated by the observations of Hertzer et al. (2022) [41].

We found a significant decrease in creatinine values in treated dogs, although no dogs showed pre-treatment values over the reference range. The effect of domperidone treatment on reducing serum creatinine levels was observed in a longitudinal, non-controlled study performed in 14 dogs with canine leishmaniosis and chronic kidney disease (CKD) [7]. A significant serum creatinine decrease was seen in nine dogs, but their proteinuria did not change at the end of the six-month follow-up [7]. A case-control study of the same research group evaluated the effect of domperidone (two courses of treatment for 30 days, three months apart) on slowing the progression of mild forms of CKD in dogs infected by *L. infantum* and fed with a renal diet [22]. At the end of the study, creatinine concentration values did not differ from those at the time of enrollment in 12 treated dogs, while a significant increase was seen in 10 controls [22]. Additionally, the concentration of serum symmetric dimethylarginine was significantly reduced after both 7 and 11 months in treated dogs, while it was significantly reduced after 7 months only in the control group [22]. A small number of dogs have been included in the above-mentioned investigations as well as in the present study; however, these field data confirm the role of DA-2 antagonists in the glomerular filtration rate of dogs and deserve to be further investigated [7,22,42].

We consider the results obtained from this study preliminary due to the small number of dogs included, particularly for investigating the ECG traces at the drug maximum peak time. Moreover, a limitation is given by the absence of a complete clinicopathological evaluation of enrolled dogs, with particular reference to liver function and liver-specific enzymes, as domperidone bioavailability is influenced by the hepatic detoxication process [20]. Studies on a larger number of dogs can investigate the variability observed in QTc changes and factors that could lead to prolonged QTc values of clinical relevance.

However, we suggest that in dogs that require treatments with domperidone, a specific risk assessment should be preliminarily obtained. Apart from the collection of a complete history, including other drugs administered, a thorough clinical examination of cardiological, electrolyte, and hepatic status would improve the safety of domperidone use.

## 5. Conclusions

Significant changes compared to the pre-treatment observations were found at the end of a four-week treatment of dogs with domperidone. The longer QTc interval measured in a majority of dogs, although within the reference range, supports the need for further investigations into the possible cardiotoxicity of this drug.

## Figures and Tables

**Table 1:** Signalment, *L. infantum* IFAT titers, corrected QT (QTc), heart rate, electrolytes (Na = sodium, K = potassium, and Cl = chloride), and renal parameters (BUN = blood urea nitrogen; Cr = creatinine) of dogs enrolled in study 1. Reference intervals for each parameter are in brackets.

Dog	Sex	Age Months	Breed	Body Weight (Kg)	*L. infantum* IFAT		QTc (150–240 ms)		Heart Rate (70–160 bpm ^A;^ 60–140 bpm ^B^)		Na (143–156 mmol/L)		K (3.5–5.5 mmol/L)		Cl (100–120 mmol/L)		BUN (14–42 mg/dL)		Cr (0.5–1.5 mg/dL)	
					Pre	Post	Pre	Post	Pre	Post	Pre	Post	Pre	Post	Pre	Post	Pre	Post	Pre	Post
1.1 ^A^	M	24	Cross-breed	20	1280	640	162.7	189.2	122	125	145.7	146.2	4.67	4.62	107.9	114.7	19	11	1.2	0.9
1.2 ^A^	M	60	Cross-breed	20	160	320	189.2	207.2	125	87	146.5	147	4.6	4.5	109.4	117	18	16	1.3	1
1.3 ^A^	M	24	Cross-breed	20	neg	neg	216.8	220	83	113	144.3	145.3	4.16	4.24	106	113.5	20	16	1.4	1.1
1.4 ^A^	M	60	Cross-breed	20	320	320	167.2	190	133	125	148.8	148.1	4.23	4.52	108.3	114.8	14	11	1.3	1
1.5 ^A^	M	60	Cross-breed	20	160	neg	175.6	203.3	153	111	144.9	144.8	4.59	4.51	107.5	112.3	21	17	1.3	0.9
1.6 ^A^	F	36	Cross-breed	20	neg	neg	170.8	210.5	136	78	146.5	143.5	4.69	4.05	109.9	115.5	20	14	1.5	1.1
1.7 ^A^	M	48	Cross-breed	20	320	160	173.9	186.7	157	250	146.3	144.9	4.85	4.31	109.4	113.2	13	12	1.4	0.9
1.8 ^A^	M	72	Cross-breed	20	160	80	210.2	190.6	150	142	143.1	146.4	4.6	4.62	105.6	109.8	15	15	1.4	1.1
1.9 ^A^	M	72	Cross-breed	20	neg	neg	200	204.6	85	87	145.2	145.9	4.31	4.19	109.6	115.5	12	14	0.9	0.9
1.10 ^A^	F	48	Italian pointer	20	2560	640	216.9	210.7	95	114	148.7	147.4	4.07	4.17	108.5	114.4	20	8	1.1	0.9
1.11 ^A^	M	48	Cross-breed	15	2560	1280	213.6	197.6	132	114	149.9	153.4	4.69	5.11	114.5	115.5	14	18	1.1	1.1
1.12 ^A^	M	48	AST	25	160	160	196.9	198.9	137	126	150.3	150.1	3.91	4.32	111.3	111.8	9	9	1.2	1.2
1.13 ^A^	F	72	Siberian husky	25	neg	neg	179.3	187.2	97	105	148.6	148	4.84	4.5	114.3	113.5	9	13	1	1.1
1.14 ^A^	M	84	English pointer	23	neg	neg	240	240.4	96	108	149.7	145.6	4.81	4.41	111.3	114.5	15	15	0.9	0.7
1.15 ^B^	F	48	CSD	40	80	neg	203.6	213.8	159	119	149	145.5	3.84	4.55	112.8	111.9	13	12	1.1	1.2
1.16 ^A^	M	108	Siberian husky	25	neg	neg	201.6	198.3	82	93	ne	148.2	ne	4.74	ne	112.3	ne	16	ne	1
1.17 ^B^	M	48	CSD	40	neg	neg	208.4	236.9	113	89	150	151.2	4.23	4.51	115.1	115.6	14	14	1.4	1.4

^A^ = standard breed; ^B^ = giant breed; ne = not evaluated; bpm = beats per minute; AST = American Staffordshire terrier; CSD = Caucasian shepherd dog.

**Table 2:** Study 1: pre- and post-treatment descriptive statistics of QTc, heart rate, and blood parameter values and *p* values of Wilcoxon matched-paired signed rank test or paired *t*-test comparisons.

Parameter (RI)	Pre-Treatment	Post-Treatment	*p*
QTc * (150–240 ms)	Mean: 195.4 SD: ±21.87	Mean: 205.1 SD: ± 16	0.0292
Heart rate (70–160 bpm ^sb^; 60–140 bpm ^gb^)	Median: 125 Min: 82 Max: 159 25th: 95.5 75th: 143.5	Median: 113 Min: 78 Max: 250 25th: 91 75th: 125	0.4655
Na (143–156 mmol/L)	Mean: 147.3 SD: ±2.298	Mean: 147.1 SD: ±2.531	0.6292
K (3.5–5.5 mmol/L)	Mean: 4.443 SD: ± 0.3344	Mean: 4.463 SD: ± 0.2487	0.9787
Cl * (100–120 mmol/L)	Mean: 110.1 SD: ± 2.92	Mean: 113.9 SD: ± 1.826	<0.0001
BUN (14–42 mg/dL)	Mean: 15.38 SD: ± 3.879	Mean: 13.59 SD: ± 2.785	0.0861
Cr * (0.5–1.5 mg/dL)	Mean: 1.219 SD: ± 0.187	Mean: 1.029 SD: ± 0.1611	0.0014

* = significant difference; RI = reference interval; QTc = corrected QT interval; bpm = beats per minute; Na = sodium; K = potassium; Cl = chloride; BUN = blood urea nitrogen; Cr = blood creatinine; ^sb^ = standard breeds; ^gb^ = giant breeds.

## Data Availability

The data set analyzed for the current study is available from the corresponding author upon reasonable request.
